# Evaluating the Impact of Nitrogen Application on Growth and Productivity of Maize Under Control Conditions

**DOI:** 10.3389/fpls.2022.885479

**Published:** 2022-05-18

**Authors:** Hafiz Mohkum Hammad, M. Shakeel Chawla, Rashid Jawad, Asma Alhuqail, Hafiz Faiq Bakhat, Wajid Farhad, Faheema Khan, Muhammad Mubeen, Adnan N. Shah, Ke Liu, Matthew T. Harrison, Shah Saud, Shah Fahad

**Affiliations:** ^1^Department of Agronomy, Muhammad Nawaz Shareef University of Agriculture, Multan, Multan, Pakistan; ^2^Department of Environmental Science, COMSATS University Islamabad, Vehari, Pakistan; ^3^Army Public School and College Mailsi Garrison, Mailsi, Pakistan; ^4^Department of Horticulture, Ghazi University, Dera Ghazi Khan, Pakistan; ^5^Chair of Climate Change, Environmental Development and Vegetation Cover, Department of Botany and Microbiology, College of Science, King Saud University, Riyadh, Saudi Arabia; ^6^Department of Agronomy, University College of Dera Murad Jamali Naseerabad, Lasbela University of Agriculture, Water and Marine Sciences, Uthal, Pakistan; ^7^Department of Agricultural Engineering, Khwaja Fareed University of Engineering and Information Technology, Rahim Yar Khan, Pakistan; ^8^Tasmanian Institute of Agriculture, University of Tasmania, Burnie, TAS, Australia; ^9^College of Life Science, Linyi University, Linyi, China; ^10^Hainan Key Laboratory for Sustainable Utilization of Tropical Bioresource, College of Tropical Crops, Hainan University, Haikou, China; ^11^Department of Agronomy, Faculty of Agricultural Sciences, The University of Haripur, Haripur, Pakistan

**Keywords:** best management practices, climate variability, maize yield production, protein content, nitrogen

## Abstract

Climatic conditions significantly affect the maize productivity. Among abiotic factors, nitrogen (N) fertilizer and temperature are the two important factors which dominantly affect the maize (*Zea mays* L.) production during the early crop growth stages. Two experiments were conducted to determine the impact of N fertilizer and temperature on the maize growth and yield. In the first experiment, the maize hybrids were screened for their sensitivity to temperature variations. The screening was based on the growth performance of the hybrids under three temperatures (*T*_1_ = ambient open-air temperature, *T*_2_ = 1°C higher than the ambient temperature, and *T*_3_ = 1°C lower than the ambient temperature) range. The results showed that an increase in temperature was resulted less 50% emergence and mean emergence (4.1 and 6.3 days, respectively), while emergence energy and full emergence were higher (25.4 and 75.2%, respectively) under the higher temperature exposure. The results showed that Syngenta 7720 and Muqabla S 25W87 were temperature tolerant and sensitive maize hybrids, respectively. The second experiment was carried out to study the response of the two selected maize hybrids (Syngenta 7720 and Muqabla S 25W87) to four N fertilizer applications. The results revealed that the maximum N use efficiency (19.5 kg kg^−1^) was achieved in maize hybrids with low N application (75 kg N ha^−1^ equivalent to 1.13 g N plant^−1^). However, the maximum maize grain yield (86.4 g plant^−1^), dry weight (203 g plant^−1^), and grain protein content (15.0%) were observed in maize hybrids that were grown with the application of 300 kg N ha^−1^ (equivalent to 4.52 g N plant^−1^). Therefore, it is recommended that the application of 300 kg N ha^−1^ to temperature tolerant maize hybrid may be considered best agricultural management practices for obtaining optimum maize grain yield under present changing climate.

## Introduction

The maize crop is ranked as the third major cereal crop after wheat and rice and similarly, it is the third most widely grown cereal crop in Pakistan (Hussain et al., 2011). The maize production is highly dependent on the climatological and pedological conditions and it is necessary to determine how changes in climate and soil conditions can maintain or improve the production. The meteorological parameters determined that local climate conditions have a great deal on agricultural productivity. Considering the possible climate change and its impact on regional agricultural systems are critical. In recent years, climate change has been affecting the global agricultural productivity and precisely these impacts are more toward the negative side of crop production (Joshi et al., 2015; Boonwichai et al., 2019; Zhang et al., 2020). Soil is another key factor in determining the plants growth and their productivity (Rodrigo-Comino et al., 2018). Soil characteristics are critical and require for the precise calculations of the number of fertilizers to be supplied, and N is the key element due to its impact on the plant water relations and biota in the rhizosphere (Basso et al., 2020; He et al., 2020).

The quality of grain crops and crop yield is strongly dependent on the N fertilizer. Numerous researchers reported that N fertilizers application generally has positive and significant impacts on the crop growth and yield (Gasim, 2001; Amanullah et al., 2016). The maize plant characteristic such as leaf number per plant is increased by the application of N which improved plant height (Akintoye, 1996) by increasing the distance among the internodes and length of the internodes (Gasim, 2001). Therefore, the application of N fertilizer is also good for increasing the height, leaf area, and stem diameter as well as the fresh and dry yields of maize (Koul, 1997). Although only small amounts of irrigation water and N fertilizers are required during early growth stages (Chen et al., 2020), higher concentration of N at that time in the root zone is beneficial for achieving the high-crop yields (Ritchie et al., 1993; Hammad et al., 2018). As N is an important source of nutrition but its losses during crop growth (Wang et al., 2019) not only results in N deficiency but also negatively affects the environment (Sutton et al., 2013).

The farming communities are not well familiar about the interaction of N fertilizer with other crop input sources especially temperature which are affecting the final grain yield. Similarly, N use efficiency (NUE) can be influenced with changes in ambient temperature and N application rates (Zhang et al., 2022). So, optimum utilization of N fertilizer by a plant is a major factor for good crop production (Sinclair and Horie, 1989; Yousaf et al., 2016). An optimum supply of N fertilizer increases total biomass production and crop yield but at lower N fertilizer supply, plant dry matter accumulation of reproductive parts decreases, resulting in the lower grain yield (Monneveux et al., 2005). Hence, N fertilizer management is a crucial need of the time while considering the enigma of global food insecurity to ensure adequate grain crop production across the World (Thompson et al., 2015).

A decrease in the losses of N fertilizer from the agricultural lands is a hot topic worldwide among the agricultural researchers. Besides, N optimization ensures the soil environmental safety. As the climate change (temperature) and N fertilizer effects on maize hybrids potential yields, however, interaction among the two critical factors, i.e., temperature with N utilization has not been explored yet especially under semi-arid environments. Therefore, this study was planned for the following objectives (i) to determine the temperature effect on the growth of maize hybrids and (ii) to optimize N fertilizer dose for the local maize hybrids under the changing climate.

## Materials and Methods

### The Design and Treatment of First Experiment

**Figure 1:**
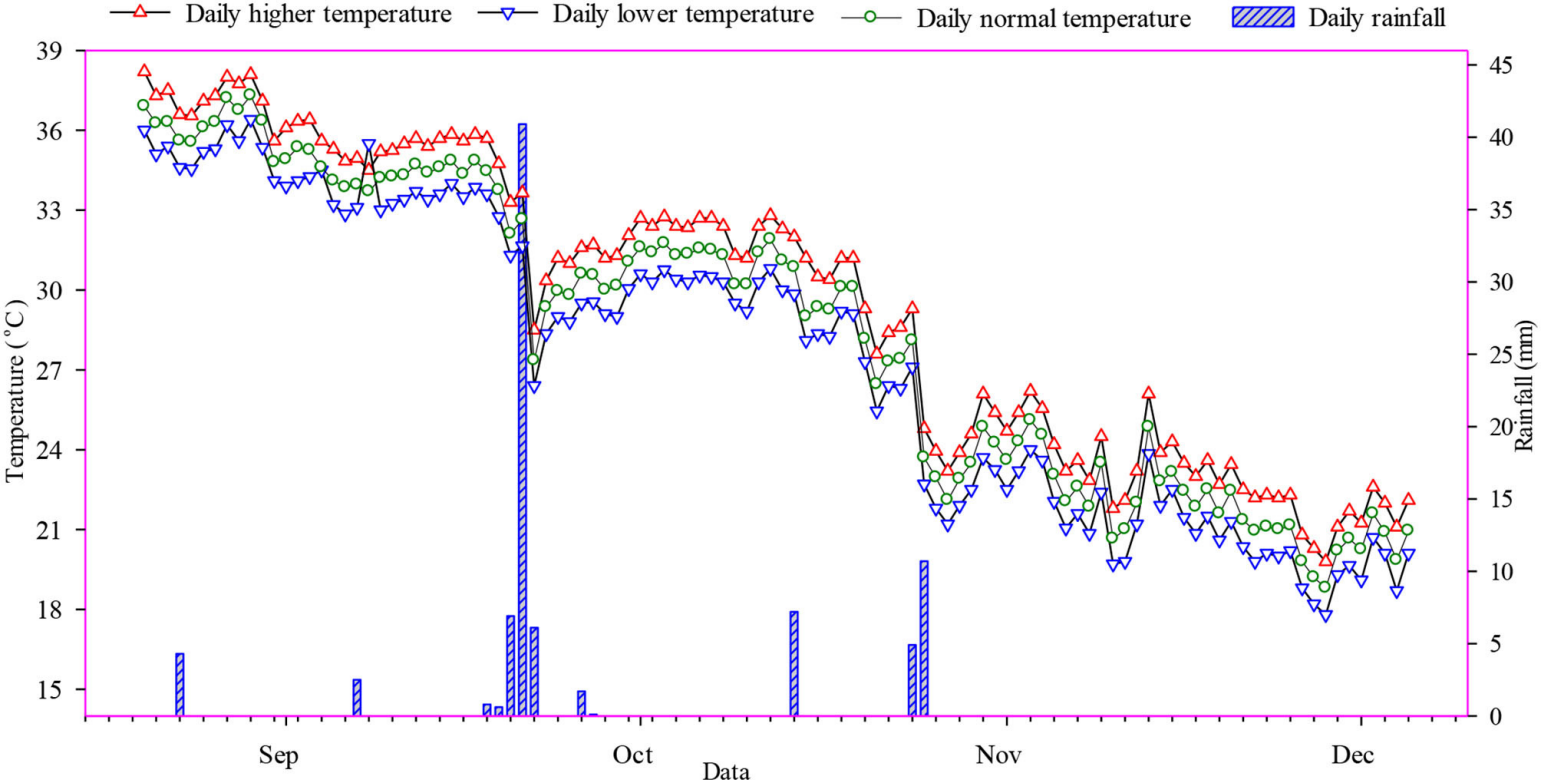
Daily temperature and rainfall data of experimental site during the growing season.

A pot experiment was conducted in glasshouse at COMSATS University Islamabad, Vehari Campus to study temperature effects on maize growth and yield. Soil analysis was carried out for determining NPK ratio in the soil. Prior to the experiment, the germination test was conducted to find out the seed germination percentage of maize hybrids. The experiment objective was to screen the 10 maize hybrids (detail shown in Table 1) against temperature sensitivity. These hybrids were grown under three different temperatures (*T*_1_ = Open-air ambient temperature, *T*_2_ = 1°C higher than ambient temperature, and *T*_3_ = 1°C lower than ambient temperature). The higher and lower temperatures were continually maintained in the glasshouse. A thermometer was placed among the pots for measuring the temperature. The completely randomized design with factorial arrangement having three replicates was used during the study. Two maize hybrids (H_1_ = heat tolerant and H_2_ = heat sensitive) were screened from the experiment on basis of germination and growth characteristics. Furthermore, a detail on daily open-air meteorological and green house data of experimental location is shown in Figure 1.

**Table 1:** Effects of various temperatures on the emergence of different maize hybrids.

**Treatment**	**50% Emergence (day)**	**SDE**	**Mean emergence (day)**	**SDE**	**Energy emergence (%)**	**SDE**	**Full emergence (%)**	**SDE**
*T*_1_	5.0	±0.7	6.8	±1.1	23.4	±2.1	70.3	±5.5
*T*_2_	4.1	±0.8	6.3	±0.9	25.4	±2.8	75.2	±5.3
*T*_3_	6.2	±0.7	7.4	±1.2	21.0	±1.8	66.9	±6.5
Significance	*P <* 0.01		*P <* 0.031		*P <* 0.01		*P <* 0.001	
LSD 5%	0.94		0.69		2.20		1.82	
H_1_	4.9	±0.6	6.3	±1.2	23.2	±1.8	68.0	±4.2
H_2_	5.0	±0.9	6.9	±1.1	22.0	±2.2	69.6	±5.7
H_3_	3.9	±0.6	5.7	±1.0	34.9	±1.9	82.4	±7.6
H_4_	4.9	±0.6	6.6	±1.2	20.9	±2.7	77.1	±4.9
H_5_	5.4	±0.4	6.7	±1.0	22.3	±1.9	72.8	±5.1
H_6_	5.0	±0.9	6.9	±1.2	20.2	±1.2	78.8	±5.7
H_7_	6.8	±0.9	9.1	±0.9	21.1	±3.9	42.3	±5.6
H_8_	4.9	±0.7	6.3	±1.4	21.4	±2.6	71.6	±7.4
H_9_	5.1	±1.0	7.0	±1.1	23.8	±2.3	72.6	±4.9
H_10_	5.1	±0.6	6.9	±1.0	22.8	±2.1	72.8	±6.6
**Mean**	5.1		6.8		23.3		70.8	
Significance	*P <* 0.001		*P <* 0.001		*P <* 0.01		*P <* 0.01	
LSD 5%	0.60		0.65		2.27		5.88	
CV	12.42		10.08		10.32		8.79	

*Values are given as means ± standard error of the means (n = 4). Means with in columns sharing different letters vary significantly at P ≤ 0.05.*

*T_1_= Normal open air temperature, T_2_ = 1°C higher temperature than normal, T_3_ =1°C lower temperature than normal.*

*H_1_ = Syngenta 6621, H_2_= Syngenta 6654, H_3_ = Syngenta 7720, H_4_ = Pioneer 30 Y 87, H_5_ = SohniDharti 626, H_6_ = ICI 339, H_7_ = Muqabla Seed 25 W 87, H_8_ = Monsento 6714, H_9_ = Rustam 1, H_10_ = Pak Ufghi.*

*LSD, Least significance difference.*

*CV, Coefficient of variance.*

*SDE, Standard Error, n = 4*.

### Observation

The experimental plants data were observed after each hour. The number of seeds emergence were noted according to the handbook of the Association of Official Seed Analysis (AOSA). (1990). Time taken to 50% seedlings (E_50_) emergence was calculated according to the formula of Coolbear et al. (1984) modified by Farooq et al. (2005).


(1) $${\text{E}}_{\text{50 }}=\;t_{i}+\left[\frac{{}^{N}\text{​​}╱\text{​}{\text{​}}_{2}\;-n_{i}}{n_{j}-n_{i}}\right]\;\left(t_{j}-t_{i}\right)$$

Mean emergence time (MET) was calculated according to the equation of Ellis and Roberts (1981).


(2) $$MET=\frac{\sum Dn}{\sum n}$$

Energy of germination was recorded at 4th day after planting. It was the percentage of emerged seedlings 4 days after planting relative to the total number of seeds tested (Farooq et al., 2006).


(3) $$EE\;\left(\%\right)=\frac{No.\;of\;seedlings\;emerged\;4\;days\;after\;sowing}{Total\;no.\;of\;seeds\;sown}\times \;100$$

The plants were harvested at maturity. The stem length (cm) was measured from base to tip of the plants. The plant shoots were cut from the pot and the soil was washed with water on iron grille to retrieve the roots. The root length (cm) was also measured from plant base to the root tip. The plants were separated into roots, shoots, and leaves. The plant each fraction was oven dried separately at 70°C till constant weight. The dry weight (DW) of roots and shoots was measured in grams (g).

### The Design and Treatments of Second Experiment

The two maize hybrids (H_1_ = Syngenta 7720 and Muqabla S H_2_ =25W87 temperature tolerant and sensitive, respectively) selected from the first experiment were tested under four different N rates with one control treatment (*N*_1_ = 0, *N*_2_ = 1.13 g plant^−1^ equal to 75 kg N ha^−1^, *N*_3_ = 2.26 g plant^−1^ equal to 150 kg N ha^−1^, *N*_4_ = 3.39 g plant^−1^ equal to 225 kg N ha^−1^, and *N*_5_ = 4.52 g plant^−1^ equal to 300 kg N ha^−1^). The completely randomized design with factorial arrangement having four replicates was used during this study.

### Plant Sampling

Plant height (cm) was measured with the help of scale from base to tip of the plants at maturity. At physiological maturity, plants were extracted from the pot and the soil was washed with the water pressure on iron grille. The root length (cm) was measured with the help of scale from plant base to root tip. The harvested plants were thrashed manually for measuring the thousand grain weight and total yield. Thousand grains were taken from each plant and sun dried up to standard moisture content in the grains. The total grains and dry matter of each plant were calculated as g plant^−1^. The Micro-Kjeldhal method (Helrich, 1990) was used for determining the N contents in grains and then the crude protein contents were determined by using the following formula


(4) Crudeprotein=N× 6.25

The NUE was calculated by the formula derived by Dobermann (2007).


(5) $$NUE=\frac{\left(Y-Y^{{}^{\circ}}\right)}{F}$$

where, *F* is the amount of N fertilizer (kg ha^−1^), *Y* is the crop yield (kg ha^−1^) with N application, and *Y*° is the crop yield (kg ha^−1^) without application of N fertilizer.

All biological processes in plant respond to temperature, and all responses can be summarized in terms of cardinal temperatures (a base temperature). Therefore, the thermal time (growing degree days) of plant was calculated according to Gallagher et al. (1983) by using the following formula:


(6) $$Tt=\frac{\Sigma (T\max+T\min)}{2}-Tb$$

where Tb is cardinal or base temperature taken as 8°C for maize (FAO, 1978).

### Statistical Analysis

The effects of treatments on the studied variables were analyzed with ANOVA using the SAS statistical software. The least significant difference test was used for comparing the means of the treatments when the *F*-values were significant at *p* 0.05 (SAS Institute, 2004). The interaction among the factors (temperature, maize hybrids, and nitrogen [N]) was non-significant, so the results of the factors were described separately.

## Results

### First Experiment

Time taken to 50% emergence of seedling was significantly affected under different temperature conditions and the hybrids showed different response to the temperature to initiate 50% emergence (Table 1). The maize hybrids took minimum time (4.1 day) for 50% emergence when they were subjected to ≈1°C higher temperature (*T*_2_) than the ambient temperature (*T*_1_) while the maximum time (6.2 day) to exhibit 50% emergence was taken by the maize seeds that were kept under *T*_3_ (≈1°C less from normal temperature). Similarly, among the maize hybrid, minimum time (3.9 day) taken to 50% emergence was observed in H_3_ (Syngenta 7720) while the hybrid H_7_ (Muqabla S 25W87) took maximum time (6.8 day) for 50% emergence.

Time taken to mean emergence of maize was significantly influenced by the exposure of crop to various temperatures (Table 1). Maize grown in approximately one-degree higher temperature (*T*_2_) from normal temperature (*T*_1_) showed significantly lower time (6.3 day) for the plant mean emergence. Precisely evaluating the performance of subjected maize hybrids, the minimum time (5.7 day) taken for mean emergence was observed in H_3_ (Syngenta 7720) and the maximum time (9.1 day) was observed in H_7_ (Muqabla S 25W87).

The results showed (Table 1) that the exposure of maize seeds to various temperatures significantly influenced the time taken by emergence energy of various maize hybrids. The maize grown under *T*_2_ treatment showed the maximum emergence energy (25.4%) and the minimum emergence energy (21.0%) was observed in maize hybrids that were kept under *T*_3_. Maximum emergence energy (34.9%) among the hybrids was observed in H_3_ (Syngenta 7720) while less emergence energy (21.1%) was observed in H_7_ (Muqabla S 25W87).

In similar terms, the time taken for full emergence of maize hybrids was also affected by various temperatures (Table 1). Seeds exposed to approximately one-degree higher (*T*_2_) temperature from ambient showed higher emergence (75.2%). The minimum time (66.9%) taken to full emergence was observed in maize hybrids that were grown in *T*_3_ unit. The hybrids used in this study showed significant response to the temperature for time taken to full emergence. The maximum full emergence (82.4%) was observed in H_3_ (Syngenta 7720) and the minimum time taken for full emergence (42.3%) was recorded in H_7_ (Muqabla S 25W87).

The stem length is an important parameter, and the results showed that the seedlings exposed to various temperatures significantly differ in stem length (Table 2). The maximum stem length (102.4cm) was recorded in maize hybrid that was grown under the ambient conditions. However, among the maize hybrid, a significant difference was observed for stem length. The maximum stem length (111.6 cm) was observed in H_3_ (Syngenta 7720), while the minimum stem length (78.4 cm) was recorded in H_7_ (Muqabla S 25W87).

**Table 2:** Effects of various temperatures on the different characteristics of maize hybrids.

**Treatment**	**Stem length (cm)**	**SDE**	**Plant dry weight (g)**	**SDE**	**Root length (cm)**	**SDE**	**Root dry weight (g)**	**SDE**
*T*_1_	102.4	±12.5	69.9	±6.5	54.8	±6.3	44.7	±5.6
*T*_2_	94.4	±12.5	61.0	±6.4	50.0	±6.4	38.1	±5.0
*T*_3_	83.4	±9.7	50.2	±7.2	43.3	±4.7	33.8	±4.2
Significance	*P <* 0.003		*P <* 0.04		*P <* 0.001		*P <* 0.01	
LSD 5%	3.6		14.1		2.1		5.1	
H_1_	86.0	±9.2	51.7	±6.1	54.1	±4.3	41.2	±5.2
H_2_	96.2	±10.6	65.6	±5.6	42.2	±5.9	33.4	±4.9
H_3_	111.6	±18.6	68.1	±7.9	65.3	±5.4	55.1	±4.7
H_4_	106.7	±13.3	69.0	±4.7	53.8	±6.5	43.2	±5.0
H_5_	104.9	±11.1	73.2	±7.5	54.7	±6.9	43.9	±5.2
H_6_	87.7	±11.4	69.8	±7.2	55.6	±5.9	46.4	±4.6
H_7_	78.4	±9.1	43.6	±6.6	28.1	±4.2	19.1	±4.7
H_8_	88.6	±11.9	56.8	±6.6	39.7	±6.9	26.1	±4.4
H_9_	94.2	±11.1	54.2	±7.8	44.4	±6.2	38.7	±5.0
H_10_	80.0	±9.5	51.7	±6.9	56.0	±5.8	41.4	±5.5
**Mean**	93.4		60.4		49.4		38.9	
Significance	*P <* 0.01		*P <* 0.008		*P <* 0.001		*P <* 0.001	
LSD 5%	5.26		4.37		1.83		2.12	
CV	6.00		7.66		5.92		5.78	

*Values are given as means ±standard error of the means (n = 4). Means with in columns sharing different letters vary significantly at P ≤ 0.05.*

*T_1_ = Normal open air temperature, T_2_= 1°C higher temperature than normal, T_3_= 1°C lower temperature than normal.*

*H_1_ = Syngenta 6621, H_2_ = Syngenta 6654, H_3_ = Syngenta 7720, H_4_ = Pioneer 30 Y 87, H_5_ = Sohni-Dharti 626, H_6_ = ICI 339, H_7_ = Muqabla Seed 25 W 87, H_8_ = Monsento 6714, H_9_ = Rustam 1, H_10_ = Pak Ufghi.*

*LSD, Least significance difference.*

*CV, Coefficient of variance.*

*SDE, Standard Error, n = 4*.

The plant DW is an important characteristic for determining the plant biomass accumulation. The results showed that the various temperatures distinctively influenced the plant DW of maize hybrids (Table 2). The maximum plant DW (69.9 g plant^−1^) was observed in maize grown under *T*_1_; however, maize hybrids that were kept under *T*_3_-exhibited minimum plant DW (50.2 g plant^−1^). Among the hybrids, the maximum plant DW (73.2 g plant^−1^) was recorded in H_5_ (Sohni-Dharti 626) which was statistically similar with maize hybrid H_4_, while the minimum plant DW (43.6 g plant^−1^) was recorded in the H_7_ (Muqabla S25W87).

The results showed that various temperatures significantly influenced the root length of maize (Table 2). The maximum root length (54.8 cm) was recorded in maize that was kept under the ambient temperature and the results were showed the root significantly decreased by decreasing the temperature. The root length was significantly varied among the maize hybrid and the maximum root length (65.3 cm) was observed in H_3_ (Syngenta 7720), while the minimum root length (28.1 cm) was recorded in H_7_ (Muqabla S25W87).

The root DW is an another important parameter that was evaluated under our prescribed temperature ranges in this study. The results showed that the root DW of maize hybrids was also affected by different temperature regimes (Table 2). The maximum root DW (11.18 g plant^−1^) was observed in maize hybrid which was grown under the ambient temperature. In case of different hybrids, the maximum root DW (13.78 g plant^−1^) was observed in H_3_ (Syngenta 7720) and the minimum plant DW (4.78 g plant^−1^) was recorded in H_7_ (Muqabla S25W87).

The results of first experiment showed the maize hybrid Syngenta 7720 was temperature tolerant while the hybrid Muqabla S25W87 was temperature sensitive. These two selected hybrids were evaluated with four N fertilizer rates including one control treatments during the second experiment.

### Second Experiment

The results showed temperature tolerant maize hybrid (Syngenta 7720) attained the higher stem length (161 cm) as compared to temperature sensitive maize hybrid (Muqabla S25W87). The application of N was significantly affected the plant growth. There was a linear relationship between plant height and N application rates during the study (Table 3). The maximum plant height (191 cm) was observed by the application of 300 kg N ha^−1^ while the minimum plant height (107 cm) was recorded in the control treatment. The application of N fertilizer was enhanced 78% higher stem length when compared with the control treatment (191 vs. 107).

**Table 3:** Effect of nitrogen (N) application on temperature tolerant (H_1_) and sensitive (H_2_) maize hybrid growth and yield.

**Treatment**	**Stem length (cm)**	**SDE**	**Thousand grain weight (g)**	**SD**	**Grain yield (g plant^**−1**^)**	**SDE**	**Total bio mass (g plant^**−1**^)**	**SDE**
H_1_	161.5 a	±32.3	242.3 a	±28.3	75.5 a	±8.1	252.1 a	±17.1
H_2_	145.7 b	±30.4	220.1 b	±30.8	63.8 b	±6.1	227.4 b	±17.1
Significance (*P*)	<0.01		<0.001		<0.0001		<0.0001	
LSD 5%	5.05		7.17		5.13		6.35	
N_1_	107.3 e	±11.9	190.5 d	±14.5	43.4 d	±4.4	174.0 e	±14.73
N_2_	139.8 d	±15.2	215.6 c	±16.5	64.1 c	±14.6	219.0 d	±24.22
N_3_	153.6 c	±12.1	233.3 b	±20.9	73.8 bc	±8.5	250.0 c	±18.41
N_4_	176.3 b	±11.4	251.0 a	±17.8	80.4 ab	±9.3	266.0 b	±19.33
N_5_	191.0 a	±13.9	265.5 a	±15.5	86.4 a	±9.7	289.8 a	±19. 9
**Mean**	153.6		231.18		69.60		239.8	
Significance (*P*)	<0.001		<0.01		<0.001		<0.0001	
LSD 5%	11.353		16.12		11.53		14.3	
CV	6.1		5.8		11.4		5.1	
Linear	^**^		^**^		^**^		^**^	
Quadratic	^*^		ns		^**^		^*^	
Cubic	ns		ns		ns		ns	

*Values are given as means ±standard error of the means (n = 4). Means with in columns sharing different letters vary significantly at P ≤ 0.05.*

*H_1_ = Syngenta 7720, H_2_ = Muqabla S25W87, N_1_= control, N_2_= 75, N_3_= 150, N_4_= 225 and N_5_= 300 kg ha^−1^, LSD = Least significance difference, CV = Coefficient of variance,*

* *Significant at the 0.05 probability level.*

** *Significant at the 0.01 probability level, ns = Non Significant.*

*SDE, Standard Error*.

The thousand grain weight is an important maize characteristic which plays a significant role for determining the maize yield. The temperature tolerant maize hybrid (H_1_) showed comparatively higher 1000 grain weight (242 g) as compared to temperature sensitive maize hybrid (H_2_) which suggests that the increasing trend of temperature might be a factor in decreasing the maize 1000 grain weight as increasing temperature decrease the growing degree days of the plant (Table 3). The results showed that there was a linear relationship between N application rates and 1000 grain weight. The maximum 1000 grain weight (265 g) was observed in the N_4_ treatment (300 kg N ha^−1^). This increase was 39.4% than the control treatment (265.5 vs. 190.5) and the minimum 1000 grain weight (190 g) was recorded in the control treatment.

**Figure 2:**
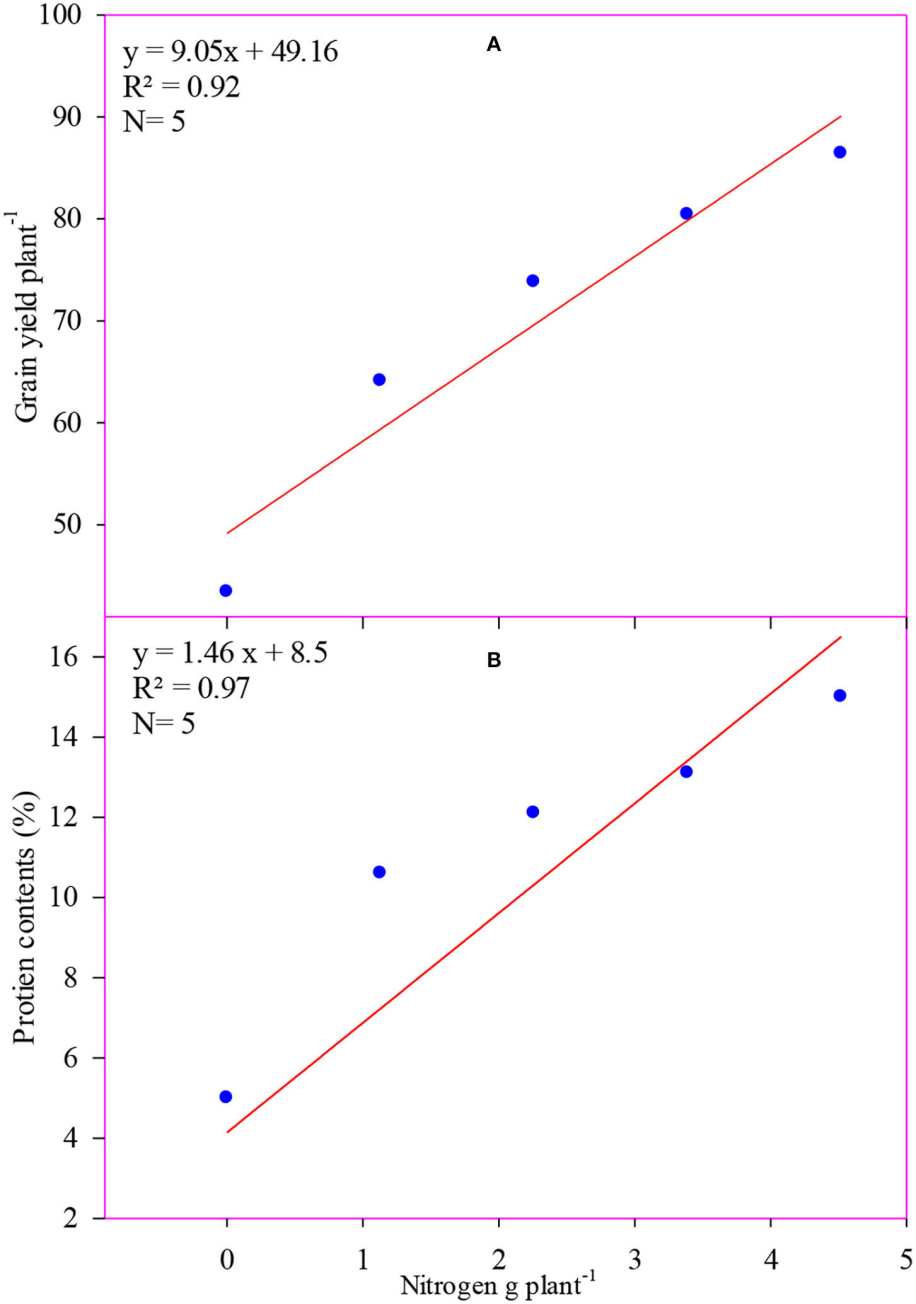
Response of grain yield **(A)** and protein contents **(B)** to nitrogen application rates.

The temperature tolerant maize hybrid (H_1_) showed higher grain yield (75.5 g plant^−1^) as compared to temperature sensitive maize hybrid (H_2_). There was a linear and quadratic relationship between N application rates and grain yield (Table 3). The maximum grain yield (86.4 g plant^−1^) was observed in the maize hybrids that were kept in N_4_ treatment (300 kg N ha^−1^). However, the minimum grain yield (43.4 g plant^−1^) was recorded in control treatment. Maize grown in N_4_ treatment resulted in 99% higher grain yield (86.4 vs. 43.4) as compared to zero N application (control). The grain yield was significantly and positively correlated (*R*^2^ = 0.92) with the N application rates (Figure 2A).

The results showed (Table 3) temperature tolerant maize hybrid (H_1_) showed significantly higher total biomass production (252 g plant^−1^) as compared to temperature sensitive maize hybrid (H_2_). The results revealed that there was a linear relationship between total biomass production and N application. The maximum total biomass (289.8 g plant^−1^) was observed in maize that was grown in N_4_ treatment and the minimum total biomass (174 g plant^−1^) was recorded in control treatment. The application of 300 kg N ha^−1^ (N_4_) resulted in 66.5% higher total biomass production (289.8 vs. 174) as compared to the control treatment.

The temperature tolerant maize hybrid showed higher root length (61.1 cm) as compared to temperature sensitive (H_1_) maize hybrid (Table 4). In addition, the N application showed linear and quadratic effects on root length. The maximum root length (71.5 cm) was observed by the application of 225 kg N ha^−1^ (N_4_) and beyond this rate, there was no significance increase in root length and the minimum root length (47.6 cm) was recorded in control treatment (N_0_). Similarly, the temperature tolerant maize hybrid (H_1_) showed the maximum root DW (17.8 g plant^−1^) as compared to temperature sensitive maize hybrid (Table 4). In case of N application, the results showed that the root DW was increased by increasing N application rate up to 300 kg ha^−1^ (N_5_). There was a linear and cubic effect of N application rates on root DW. The maximum root DW (20.3 g plant^−1^) was recorded in the N_5_ treatment. However, the minimum root weight (13.4 g plant^−1^) was recorded in unfertilized treatment, i.e., control treatment (N_1_). The application of N 300 kg ha^−1^ (N_5_) was resulted 51% (20.3 vs. 13.4) higher root DW as compared to control treatment.

**Table 4:** Effect of N application on temperature tolerant (H_1_) and sensitive (H_2_) maize hybrid characteristic.

**Treatment**	**Root length (cm)**	**SDE**	**Root weight (g)**	**SDE**	**Protein content (%)**	**SDE**	**Nitrogen use efficiency (kg kg^**−1**^)**	**SDE**
H_1_	61.1 a	±9.23	17.8 a	±2.6	10.8 a	±5.4	13.0 a	±9.8
H_2_	55.9 b	±9.23	16.4 b	±2.5	9.9 b	±4.9	8.4 b	±5.2
Significance (*P*)	<0.001		<0.001		<0.01		<0.005	
LSD 5%	2.1163		0.54		0.37		3.1084	
N_1_	47.6 c	±5.55	13.4 d	±1.2	8.0 e	±0.1	0.0 c	±0.0
N_2_	54.7 b	±4.83	16.3 c	±1.6	10.6 d	±1.2	19.5 a	±6.7
N_3_	59.2 b	±4.80	17.0 c	±0.9	12.1 c	±0.7	13.4 ab	±3.7
N_4_	71.5 a	±6.74	18.3 b	±1.5	13.1 b	±0.8	10.9 b	±2.3
N_5_	59.5 b	±5.58	20.3 a	±1.3	15.0 a	±0.9	9.5 b	±1.5
**Mean**	58.5		17.0		11.76		10.6	
Significance (*P*)	<0.01		<0.01		<0.001		<0.01	
LSD 5%	4.76		1.21		0.83		6.99	
CV	5.62		4.90		6.52		5.25	
Linear	^**^		^**^		^**^		^**^	
Quadratic	^*^		ns		^**^		^**^	
Cubic	Ns		^**^		^**^		^**^	

*Values are given as means ±standard error of the means (n = 4). Means with in columns sharing different letters vary significantly at P ≤ 0.05.*

*H_1_ = Syngenta 7720, H_2_ =Muqabla S25W87, N_1_= control, N_2_= 75, N_3_= 150, N_4_= 225 and N_5_= 300 kg ha^−1^,*

*LSD= Least significance difference, CV = Coefficient of variance,*

* *Significant at the 0.05 probability level.*

** *Significant at the 0.01 probability level, ns, Non-Significant*.

Like other parameters, the higher protein content in grains (10.8%) was recorded in temperature tolerant maize hybrid (H_1_) as compared to temperature sensitive maize hybrid (Table 4). The results showed that the protein content in grain increased by increasing the N application rate up to 300 kg ha^−1^ (N_5_). There was a cubic effect of N application rates that was observed on the protein content in grains. The maximum protein content in grain (15.0%) was observed in the N_5_ treatment, while the minimum protein content in grain (8.0%) was recorded in the control treatment. The application of N 300 kg ha^−1^ resulted in 47% higher protein content (15.0 vs. 8.0) in grains as compared to the control treatment. The protein contents were significantly correlated (*R*^2^ = 0.97) with N application rates (Figure 2B).

The results revealed temperature tolerant maize hybrid (H_1_) attained significantly higher NUE (13.0 kg kg^−1^) as compared to H_2_ maize hybrid (Table 4). The results showed that the NUE was decreased by increasing N application rate. The maximum NUE (19.5 kg kg^−1^) was observed by the application of N at rate of 75 kg ha^−1^ (N_2_) and the minimum NUE (9.5 kg kg^−1^) was recorded in N_5_ treatment.

The crop development of second experiment is showed in Table 5 which represented calendar days and thermal time (growing degree days) from sowing to emergence. It has also been seen that the maize hybrids did not show any response to different N fertilizer application doses to thermal time from emergence to silking. However, the response was observed significant during the later crop growth stages. The temperature tolerant maize hybrid (H_1_) and higher doses of N fertilizer application attained more thermal time than temperature sensitive (H_2_) and other maize units which received lower N fertilizer rates. The application of 225 and 300 kg N ha^−1^ resulted in the maximum thermal time (1975°C days) in temperature tolerant maize hybrid and the minimum thermal time (1937°C days) was observed in control treatment (no N application).

**Table 5:** Effect of temperature and N application on crop phenology.

**Crop stages**	**Treatments**	**Calendar date**	**Calendar days**	**Thermal time** **(° C days)**
Sowing	H_1_ H_2_	24-08-15 24-08-15	– –	– –
	N_1_ N_2_ N_3_ N_4_ N_5_	24-08-15 24-08-15 24-08-15 24-08-15 24-08-15	– – – – –	– – – – –
Sowing to emergence	H_1_ H_2_	27-08-15 27-08-15	3 3	113 113
	N_1_ N_2_ N_3_ N_4_ N_5_	27-08-15 27-08-15 27-08-15 27-08-15 27-08-15	3 3 3 3 3	113 113 113 113 113
Emergence to tasseling	H_1_ H_2_	13-10-15 11-10-15	48 46	1170 1123
	N_1_ N_2_ N_3_ N_4_ N_5_	11-10-15 10-10-15 12-10-15 14-10-15 14-10-15	46 45 47 49 49	1123 1100 1146 1193 1193
Emergence to silking	H_1_ H_2_	19-10-15 18-10-15	54 53	1301 1279
	N_1_ N_2_ N_3_ N_4_ N_5_	17-10-15 17-10-15 18-10-15 20-10-15 20-10-15	52 52 53 55 55	1258 1258 1279 1324 1324
Emergence to crop maturity	H_1_ H_2_	02-12-15 30-11-15	98 96	1962 1937
	N_1_ N_2_ N_3_ N_4_ N_5_	30-11-15 30-11-15 02-12-15 03-12-15 03-12-15	96 96 98 99 99	1937 1937 1962 1975 1975

*H_1_ = Syngenta 7720 (Temperature tolerant), H_2_ =Muqabla S25W87 (Temperature sensitive).*

*N_1_= control, N_2_= 75, N_3_= 150, N_4_= 225 and N_5_= 300 kg ha^−1^*.

## Discussion

The results revealed that an increase in temperature accelerated seed germination of maize hybrids. As during the spring season under semi-arid conditions, sometime air temperature is low at initial stages from maize base temperature (8°C). The response of maize seed emergence to temperature was best with minimum cardinal temperatures ranging from 8 to 37.5°C, and optimum temperatures of 25.9 to 37.0°C (Edalat and Kazemeini, 2014). Hence, an increased temperature enhanced the germination capacity coupled with the indication that optimum temperature is the pre-requirement for the maize germination process (Meng et al., 2022). It is predicted in the scenarios, air temperature will increase due to climate change, which could limit the length of the maize-growing season if other resources are not limited (Odgaard et al., 2011).

Morroco et al. (2005) stated that the high level of deviation, in response to changing temperatures, was recorded among the maize hybrids concerning physiological and morphological characteristics of the plant, which indicated that alleles are present which can improve the plant adoptability to lower and higher temperature conditions. Being a complex phenomenon, i.e., chilling stress, cold tolerance is generally controlled with the influence of multiple genes. Hence, to understand the responses of maize to low temperature stress at emergence stage, a multidisciplinary approach comprising physiology, genetics, and molecular biology will be the best way. The result from our study revealed that an increase in temperature resulted in the fast germination mechanism in the seeds. Even though there was a slight difference of temperature among the treatments; it was still enough to create differences observable for the maize hybrids tolerance potential under varying temperature conditions. However, higher temperature at later crop growth stages especially at reproductive stage resulted in lower grain yield due to decrease in pollen development (Ji et al., 2010). Furthermore, higher temperature during the whole growing season was also resulted in early plant maturity as the plant attained required thermal time early and they have less time for dry matter accumulation through photosynthesis and nutrients from the soil (Hammad et al., 2013; Hatfield and Prueger, 2015). Khaliq et al. (2008) studied the thermal time and calendar days of maize different cultivars and varying rate of N application and they found significant effects of N fertilizer on maize hybrid. Similar effects were also observed by Hammad et al. (2013) and the authors have concluded that the N application timing and rates were affected the maize growth and development. The observed lower yield and yield components including stem and root length, root weight, and thousand grain weight in maize with one-degree higher temperature are due to the less duration of developmental phases, diminish the photosynthetic potential coupled with increased respiration, lower light interception, and pollen sterility (Cairns et al., 2013). In *T*_2_ treatment units (1° higher temperature than ambient), the pollen viability was negatively affected by the heat stress as pollen desiccation is severely affected by the surrounding air temperature (Fonseca and Westgate, 2005).

Our results were also in accordance with the study of Ordóñez et al. (2015) highlighting that higher temperature is the main reason for the reduction in maize crop yield by influencing the developmental trends in ear growth. The exposure of maize hybrids toward higher temperature (*T*_2_) at silking stages is a strong factor for lower final yield (Cicchino et al., 2010) through affecting the distribution of photosynthates among the sources and sinks in maize plants. Moreover, this source capacity is in fact influenced by a decline in carbohydrate synthesis (Barnabás and Fehér, 2008) which is due to the downscaled photosynthesis plus higher respiration rates (Ordóñez et al., 2015). In addition, the sink capacity is negatively affected by the deterioration in the synchrony of anthesis-silking phenomena that leads to decrease the ovule fertilization and increased the kernel abortion. Eventually, these impacts cause the disturbance in the process of pollination and kernel set, hence, causing severe yield losses (Edreira et al., 2011).

The crop phenology and growth notably responded to varying rate of N fertilizer. An increased rate of N fertilizer up to the optimum dose is a sound approach to attain the highest yield. The best possible development and maize grain yield were accomplished by the application of N at the rate of 300 kg N ha^−1^. The application of N increased cell counts and volume per leaf as well as it accelerated the formation of chlorophyll and increased plants biomass during the early stages of crop growth. The results were supported by the findings of Amanullah et al. (2009) and Hammad et al. (2011) who reported that the application of N fertilizer was increased the crop duration in maize.

Hammad et al. (2013) also observed that 2072°C thermal days with the application of 250 kg N ha^−1^ were required to obtain optimum maize yield. The crop phenology was influenced by temperature and N fertilizer application rate. Furthermore, it has also been evaluated that a continuous enhancement in N application is not so beneficial in term of NUE parameter (Gheysari et al., 2009). Paolo and Rinaldi (2008) concluded that NUE could be improved only at a specific N fertilizer rate. The fact that the highest water use efficiency was attained with the application of more water and N discloses that both water and N are yield-limiting factors in semiarid environments. However, mostly in developing countries, each farmer's main objective is final the economic yield of the crop. The maximum grain yield of the maize crop was achieved by the application of N up to 300 kg ha^−1^ (equivalent to 4.52 g N plant^−1^). Therefore, this rate of N should be considered as a best fertilizer management practice under the semiarid regions in Pakistan and similar regions in the rest of the World.

## Conclusion

The increase in temperature decrease the time required for germination time as well as for maturity in maize with less growing degree days which resulted in lower grain yield. The results showed that the increase in the N application rate up to 300 kg ha^−1^ (equivalent to 4.52 g N plant^−1^) resulted maximum grain yield and total dry matter production, but increase is in N application rate is not a sound strategy for attaining maximum N use efficiency. Therefore, it is concluded the applications of 300 kg N ha^−1^ by selecting temperature resilient maize hybrids should be considered a good agricultural practice for achieving optimum maize grain yield, in semiarid region of Pakistan. Furthermore, future global warming will result in a reduction in maize grain yield. Therefore, it is suggested that plant breeders policy makers should develop new temperature tolerant maize hybrids for achieving the high-crop productivity under changing climate.

## Data Availability Statement

The original contributions presented in the study are included in the article/supplementary material, further inquiries can be directed to the corresponding author/s.

## Author Contributions

HH: conceptualization and supervision. MC, RJ, AA, FK, HB, WF, MM, and AS: methodology and formal analysis. SF: writing—original draft preparation. SS, KL, MH, AA, FK, and SF: writing—review and editing. AA: funding acquisition. All authors contributed to the article and approved the submitted version.

## Conflict of Interest

The authors declare that the research was conducted in the absence of any commercial or financial relationships that could be construed as a potential conflict of interest.

## Publisher's Note

All claims expressed in this article are solely those of the authors and do not necessarily represent those of their affiliated organizations, or those of the publisher, the editors and the reviewers. Any product that may be evaluated in this article, or claim that may be made by its manufacturer, is not guaranteed or endorsed by the publisher.
